# Clinical effectiveness of restorative materials for the restoration of carious primary teeth without pulp therapy: a systematic review

**DOI:** 10.1007/s40368-022-00725-7

**Published:** 2022-07-12

**Authors:** S. Amend, C. Boutsiouki, K. Bekes, D. Kloukos, N. N. Lygidakis, R. Frankenberger, N. Krämer

**Affiliations:** 1 Department of Paediatric Dentistry, Medical Centre for Dentistry, Justus-Liebig-University Giessen, University Medical Centre Giessen and Marburg (Campus Giessen), Schlangenzahl 14, 35392 Giessen, Germany; 2 Department of Paediatric Dentistry, Medical University Vienna, University Clinic of Dentistry, Sensengasse 2a, 1090 Vienna, Austria; 3 Department of Orthodontics and Dentofacial Orthopedics, University of Bern, Freiburgstrasse 7, 3010 Bern, Switzerland; 4 Lygidakis Dental Clinic (Private Dental Practice), 2 Papadiamantopoulou str. & Vasilissis Sofias Ave, 11528 Athens, Greece; 5 Department of Operative Dentistry, Endodontology, and Paediatric Dentistry, Medical Centre for Dentistry, Phillips-University Marburg, University Medical Centre Giessen and Marburg (Campus Marburg), Georg-Voigt-Str. 3, 35039 Marburg, Germany

**Keywords:** Primary teeth, Dentine, Caries, Restorative materials, Clinical effectiveness, Systematic review

## Abstract

**Purpose:**

To systematically search the available evidence and evaluate the clinical effectiveness of restorative materials for restoration of carious primary teeth. The findings aimed to support the European Academy of Paediatric Dentistry (EAPD) guidelines development.

**Methods:**

Literature search was performed by searching 4 electronic databases for eligible randomised controlled clinical trials (RCTs) comparing restorative materials for the restoration of carious primary teeth up to December 28th, 2020. Quality assessment was performed with the revised Cochrane risk-of-bias tool for randomized trials (RoB 2).

**Results:**

Of 1685 identified articles 29 RCTs were finally deemed as eligible for inclusion. Annual failure rates were: Amalgam 1–28%; atraumatic restorative treatment 1.2–37.1%; glass-ionomer cement (GIC) 7.6–16.6%, metal-reinforced GIC 29.9%, resin-modified GIC 1.9–16.9%, high-viscosity GIC 2.9–25.6%; glass carbomer ≤ 46.2%; compomer 0–14.7%; composite resin (CR) 0–19.5%, bulk-fill CR 0–16.9%; zirconia crowns 3.3%, composite strip crowns 15%, and preformed metal crowns (Hall-Technique) 3.1%. Secondary caries, poor marginal adaptation, loss of retention, and fracture of restoration were reported as reasons for failure. Four studies were evaluated at unclear and 25 at high risk of bias. Clinical and methodological heterogeneity, and the diversity of tested materials across included studies did not allow for meta-analyses.

**Conclusions:**

Within the limitations of this systematic review, namely, the heterogeneity and the overall high risk of bias among included studies, clear recommendations based on solid evidence for the best restorative approach in primary teeth cannot be drawn. There is a need for future thoroughly implemented RCTs evaluating restorations in primary teeth to close this knowledge gap.

**Supplementary Information:**

The online version contains supplementary material available at 10.1007/s40368-022-00725-7.

## Introduction

Dental caries affects 2.4 billion adults and 621 million children worldwide (Kassebaum et al. 2015). Lancet’s Global Burden of Disease (GBD) assessment generally demonstrated oral diseases being one major challenge for global public health (Peres et al. 2019). In primary teeth, untreated dental caries is estimated to cause costs of > $532 Mio, with richer countries showing a significantly lower prevalence (Vernazza et al. 2021). The main daily work of dentists is still the restoration of carious teeth and the replacement of pre-existing restorations (Dos Santos Pinto et al. 2014; Franzon et al. 2015; Hübel and Mejare 2003; Kavvadia et al. 2004). Facing the global needs, there is a general urgent need for the treatment of primary teeth caries (Peres et al. 2019; Vernazza et al. 2021).

Traditionally, dental amalgam has had its place in paediatric dentistry worldwide (Dutta et al. 2001; Hse and Wei 1997; Sengul and Gurbuz 2015). In 2017, the parties to the Minamata Convention on mercury agreed to phase down the use of dental amalgam, especially in the dental treatment of vulnerable groups such as children, pregnant, or breastfeeding women to reduce their exposure to mercury (Minamata Convention on Mercury 2013). Another important aspect is that for both cement lining and amalgam, there may not be enough space in primary molars due to large pulps. Following these reasons, a clear paradigm shift in favour of minimally invasive preparation and restoration is clearly seen (Attin et al. 2001; Hse and Wei 1997). Consequently, adhesive restorations placed in primary dentition revealed the best clinical performance; however, their use still is time-consuming and considerably technique-sensitive (Laske et al. 2016; Opdam et al. 2014; van de Sande et al. 2013). Hence, in most of the studies considerable amounts of failures have been reported, most of them related to secondary caries (Laske et al. 2016; Opdam et al. 2014). Effective restoration of primary teeth is dependent on several factors, i.e., the compliance of the child, operator skills in both behavioural and technical management, and finally the individual properties of the chosen restorative biomaterial—in decreasing order of clinical importance (Chisini et al. 2018). Furthermore, it is evident that lower age correlates with lower restoration survival of primary tooth restorations (Chisini et al. 2018).

In relation to these patient behaviour management problems, operator skill related factors, and material properties, the ideal restorative material for primary teeth has not been found so far: Amalgam (A) is characterised by low technique-sensitivity but quite aggressive preparation requirements without actually sufficient space for this, since primary teeth are smaller and pulp is closer to the outer surface (Daou et al. 2009; Hilgert et al. 2014). Resin-based composites (CR) are minimally invasive and quite easily bonded when recent universal adhesives are employed. However, they are still considerably technique-sensitive, related to both operator’s abilities and contamination issues (Casagrande et al. 2013; Cavalheiro et al. 2020). Glass-ionomer cements (GIC) are true bulk-fill materials and, therefore, favourable per se, but GIC require undercuts such as amalgam as well and they are prone to fracture due to inferior flexural strength and fatigue characteristics (Espelid et al. 1999; Kilpatrick et al. 1995; Krämer and Frankenberger 2001). Resin-modified glass-ionomer cements (RMGIC) exhibit significantly higher flexural strengths compared to conventional GICs but they also require undercuts; their inferior wear resistance seems to be uncritical for primary teeth (Hübel and Mejare 2003; Kotsanos and Arizos 2011). Last but not least, preformed metal crowns (PMC) offer an overall good clinical performance, on the other hand they are mainly indicated for extensive carious defects in primary teeth and for cuspal coverage/better coronal seal post endodontic treatment, or the Hall-technique. Their aesthetics really are a major concern when dealing with parents in daily dental practice (Donly et al. 2020; Hutcheson et al. 2012).

The aim of the present study was to systematically review the clinical effectiveness of different restorative materials including new biomaterials for the restoration of carious primary teeth. Special focus was placed to evaluate the different failure modes related to material characteristics.

## Methods

### Protocol and registry

The present systematic review was conducted according to the updated PRISMA guidelines (Preferred Reporting Items for Systematic Reviews and Meta-Analyses) (Page et al. 2021). The review protocol was registered before review commencement in PROSPERO international prospective register of systematic reviews hosted by the National Institute for Health Research (NIHR), University of York, UK, Centre for Reviews and Dissemination (CRD42020221944).

### Review’s focused question

What is the clinical effectiveness of restorative materials including new biomaterials used for the restoration of carious primary teeth with a follow-up of at least 12 months?

### PICO(S) construct

Population: Children (regardless of sex and age) with primary, previously unrestored carious lesions in primary teeth, which were treated with a restorative approach.

Intervention: (i) Randomised controlled clinical trials (RCTs) comparing different techniques and degrees of caries removal (selective vs. complete caries removal) in combination with the same/different restorative material(s) (adhesive/compomer, adhesive/composite, glass-ionomer cement (GIC), resin-modified glass-ionomer cement (RMGIC), metal-reinforced glass-ionomer cement (MRGIC), bio-active materials (BM), amalgam, preformed metal/zirconia/composite crowns) placed as restorations in primary dentition. (ii) RCTs comparing the same approach for caries removal combined with different restorative materials (adhesive/compomer, adhesive/composite, glass-ionomer cement (GIC), resin-modified glass-ionomer cement (RMGIC), metal-reinforced glass-ionomer cement (MRGIC), bio-active materials (BM), amalgam, preformed metal/zirconia/composite crowns) placed as restorations in primary dentition.

Comparison(s): Conventional restorative approach using another technique or degree of caries removal and/or another restorative material to restore carious lesions in primary teeth.

Outcome: The primary outcomes for the clinical effectiveness of restorations in primary teeth included (i) treatment failure evaluated according to the modified USPHS criteria (Krämer et al. 1999; Roulet 1994; Ryge and Snyder 1973), and (ii) restoration quality assessed according to the following criteria: surface roughness, colour match, marginal integrity, integrity (tooth/filling), proximal contact, change of sensitivity, hypersensitivity and radiographic assessment. To assess the failure of crowns, the following outcome criteria needed to be described: Modified USPHS criteria (restoration failure, proximal contact, marginal integrity, occlusion, secondary caries (Alaki et al. 2020)) or outcome criteria such as success/major failure/minor failure (Santamaria et al. 2017).

Secondary outcomes were: (i) time until restoration failure/re-treatment, (ii) discomfort during restorative treatment or within 24 h after treatment, (iii) patient’s and/or carer’s perceptions of the restorative treatment, (iv) impact of the following factors on the clinical effectiveness of the restorative treatment: Preoperative radiograph, caries lesion depth, affected tooth surface(s), extent of carious removal, type of tooth, isolation technique, type of adhesive, type restorative material.

Study design: Randomised controlled clinical trials (RCTs).

### Inclusion criteria

The following criteria of RCTs needed to be fulfilled for inclusion:

- Children with carious lesions in primary teeth extending into dentine and requiring intervention, preferably with a description of the lesion depth.
- Vital, asymptomatic teeth with no history of pain, pulp exposure, infection, swelling and no evidence of furcal/periapical inflammation.
- Restorative treatment with different restorative materials or techniques and degrees of caries removal (selective or complete removal of carious tissue).
- Clinical follow-up of at least 12 months and a minimum of 40 restorations per group (Chisini et al. 2018).
- Evaluation of any of the following outcomes: Treatment failure (*i. e.* according to the modified USPHS criteria) or assessment of restoration quality (surface roughness, colour match, marginal integrity, integrity (tooth), integrity (filling), proximal contact, change of sensitivity, hypersensitivity, and radiographic assessment).

### Exclusion criteria

The following exclusion criteria were applied:

- Any publication not fulfilling the criteria mentioned above or not related to the aim and outcomes of the current systematic review.
- Studies that were not RCTs.
- Studies conducted in permanent teeth.
- Studies with teeth treated by vital and non-vital pulp therapy.
- Studies with a drop-out rate > 30% (Tedesco et al. 2017).

### Search strategy

The literature search was performed by one experienced researcher (DK) screening the following 4 electronic databases on December 28, 2020: MEDLINE (PubMed), EMBASE (via Ovid), Cochrane Library, and LILACS. The search strategy was adjusted to the requirements of each electronic database. Hand search was carried out to find additional studies that had not been identified during electronic database search; reference lists of included studies and of systematic reviews on restorative treatment in primary dentition were screened. No restrictions were applied to language or publication year. The search strategies of all electronic databases are presented in Appendix 1.

### Study selection

Two reviewers (CB, SA) carried out study selection independently and in duplicate in the following stages:

1. Initial screening of titles and abstracts of potential papers according to the inclusion criteria by two reviewers (CB, SA), resulting in a database for which full-text articles were retrieved. Rayyan QCRI application was used for the initial filtering of the studies (Ouzzani et al. 2016).
2. Screening by two reviewers (CB, SA) of the full-text papers identified as possibly relevant to the question of the review. Manuscripts of studies published several times were excluded if they presented identical results or data that were not within the scope of this systematic review. Only the latest published manuscript presenting relevant outcomes was included. In case of studies reporting relevant outcomes in several manuscripts, all of them were assessed for eligibility.
3. Handsearching by two reviewers (NNL, SA) in the reference lists of included papers and systematic reviews on restorative treatment in primary dentition for any missed potential papers.

Disagreements between reviewers in any stage were resolved by consensus-based discussion. Reviewers were blinded neither to author names and study sites, nor to the results of the included RCTs. A record of the study selection procedure was kept.

### Data collection

Full-text articles were obtained from or ordered through the University of Giessen library for all the initially included studies. Data from the finally selected RCTs were extracted from the full-text papers and entered into an Excel table for further analysis by two independent reviewers (CB, SA). The reviewers performed a calibration training using the first 10 studies of data extraction and risk of bias assessment. For each selected trial the following data were recorded:

- Authors, title, year of publication
- Study design (split-mouth or parallel group), country of origin
- Setting of the clinical study, follow-up intervals
- Sample size at baseline and at each follow-up > 12 months, age of participants, sex, inclusion and exclusion criteria
- Detailed description of interventions, techniques and materials used (caries risk, type of tooth, cavity class, type of anaesthesia, type of isolation, technique of caries removal, restorative technique)
- Primary and secondary outcomes (clinical and radiographic)
- Results/Findings (success scores, failure scores, reasons for failure)
- Study conclusions
- Notes (ethical approval, funding, trial registration, sample size calculation, informed consent, number and calibration of operator(s) and outcome assessor(s), randomisation technique, drop-out, limitations)

### Quality assessment of the included studies

The quality assessment of the selected RCTs was performed using the Cochrane risk-of-bias tool for randomized trials version 2 (Sterne et al. 2019). It was conducted independently by two reviewers (CB, SA) without blinding the name of authors, institutions and journals. The aim of the evaluation was to assess the effect of assignment to the intervention (the intention-to-treat effect). Disagreements between reviewers were resolved by consensus-based discussion. A third reviewer (DK) was consulted in cases, where consensus was not reached.

### Data analysis

Meta-analyses were planned to be computed if there were studies of similar comparisons reporting the same outcomes. Due to the considerable clinical and methodological heterogeneity among included studies, meta-analyses were not performed.

### Calculation of failure rates

Failure rates and annual failure rates (AFR) reported by trial authors were extracted. If Kaplan–Meier statistics had been used by the study authors to estimate survival rates of restorations, failure rates were calculated based on the data presented for survival analysis. In all other cases, parameters of reported evaluation criteria (Frencken 2009; Hickel et al. 2007; Krämer et al. 1999; Roulet 1994; Ryge and Snyder 1973) were transferred into dichotomous data (acceptable/unacceptable clinical performance of restorations) to calculate failure rates (Table 1 according to Dias et al. 2018).

**Table 1:** Dichotomy of the evaluation criteria applied in the included RCTs

Parameters for failure assessment	Modified USPHS criteria		FDI criteria		ART criteria	
	Acceptable	Unacceptable	Acceptable	Unacceptable	Acceptable	Unacceptable
Secondary caries	Alpha, Bravo	Charlie, Delta	1, 2, 3	4, 5	0, 1	C
Marginal adaptation/ integrity	Alpha, Bravo	Charlie, Delta	1, 2, 3	4, 5	0, 1	2, 5
Fractures	Alpha, Bravo	Charlie, Delta	1, 2, 3	4, 5	0, 1	3, 4
Loss of retention	Alpha, Bravo	Charlie, Delta	1, 2, 3	4, 5	0, 1	6, 7

The AFR was computed by division of the failure rate by years of follow-up and according to a formula presented by Opdam et al. (2014):

$$\left( {{1}{-}{\text{y}}} \right)^{\text{z}}\, = \,({1}{-}{\text{x}})$$.

x = total failure rate at ‘z’ years.

y = mean AFR.

In addition, reasons for failure of restorations (secondary caries, marginal adaptation, fracture/anatomical form, and loss of retention) were extracted.

## Results

### Selection of the studies

**Fig. 1:**
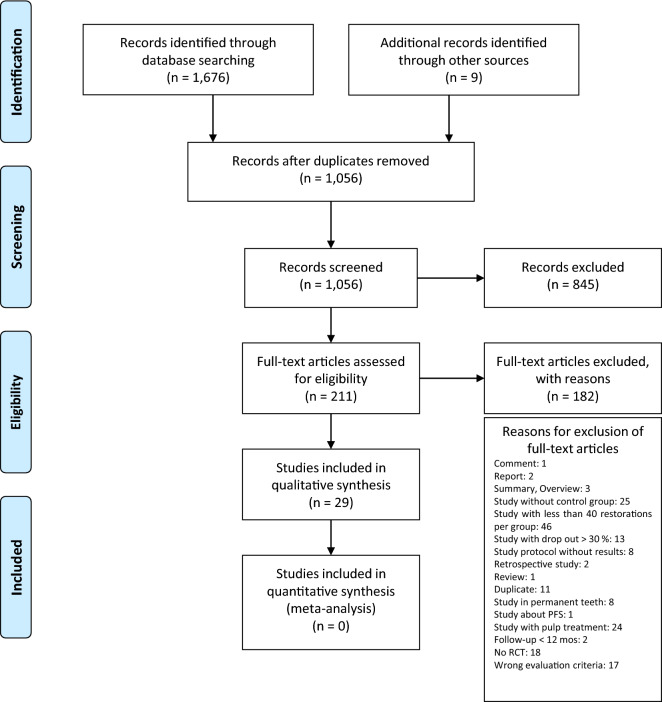
PRISMA flow diagram presenting the study selection process, the numbers of studies identified, eligible, and included in the systematic review. *PFS* pit and fissure sealing, *RCT* randomised clinial trial

Based on the selection criteria, 1,676 articles were identified through database screening and 9 additional papers were identified through other sources. Among these records, 629 duplicates were removed. Another 845 records were excluded, because the title and/or the abstract did not fulfil the inclusion criteria. 211 full-text articles were assessed for eligibility. The reasons for exclusion of 182 full-text articles are presented in Fig. 1. Thus, 29 articles remained for qualitative synthesis and no record was included in the quantitative analysis (Fig. 1).

### Characteristics of included studies

The characteristics of the 29 randomised controlled clinical trials included in this systematic review are listed in Tables 3 and 4. Among the 29 RCTs, 27 studies included primary molars, 1 study evaluated the clinical performance of crowns in primary incisors (Alaki et al. 2020), and one study included anterior and posterior primary teeth (Moura et al. 2020). Studies included between 25 and 568 children aged 1.5–11 years. All in all, 5,892 teeth distributed among 2,927 children received a restorative treatment. The follow-up period of the restorations ranged from 12 to 60 months. Seventeen studies were conducted in split-mouth design and 12 studies used a parallel group design. Seventeen of the studies were carried out in universities or dental schools, seven in a school setting, three in private dental practices, and in 2 cases the clinical setting was not reported. Nineteen papers evaluated the restoration quality by modified USPHS or Ryge criteria (Krämer and Frankenberger 2001; Krämer et al. 1999; Roulet 1994; Ryge and Snyder 1973), two used the FDI criteria (Hickel et al. 2010; Hickel et al. 2007), and 7 the criteria for ART restorations (Frencken 2009). Thirteen studies included Class-I restorations; 21 studies focused on Class-II restorations. In two trials, authors reported about crowns as restoration on vital primary teeth. In 14 studies cotton rolls were used for isolation, in 9 trials rubber dam was applied, 1 trial had a combination of both isolation methods, and in 7 papers the isolation technique was not reported. Information about the procedure of caries removal was very limited, since 19 studies gave some information on caries removal, being partial, complete or ART. Studies with ART were separately examined. Limited data was the reason why we did not further evaluate this parameter. All in all, the study designs, the treatment protocols, and the outcomes were described heterogeneously. Tables 3 and 4 list the results split by the different restorative materials or techniques used in the included RCTs.

**Table 2:**
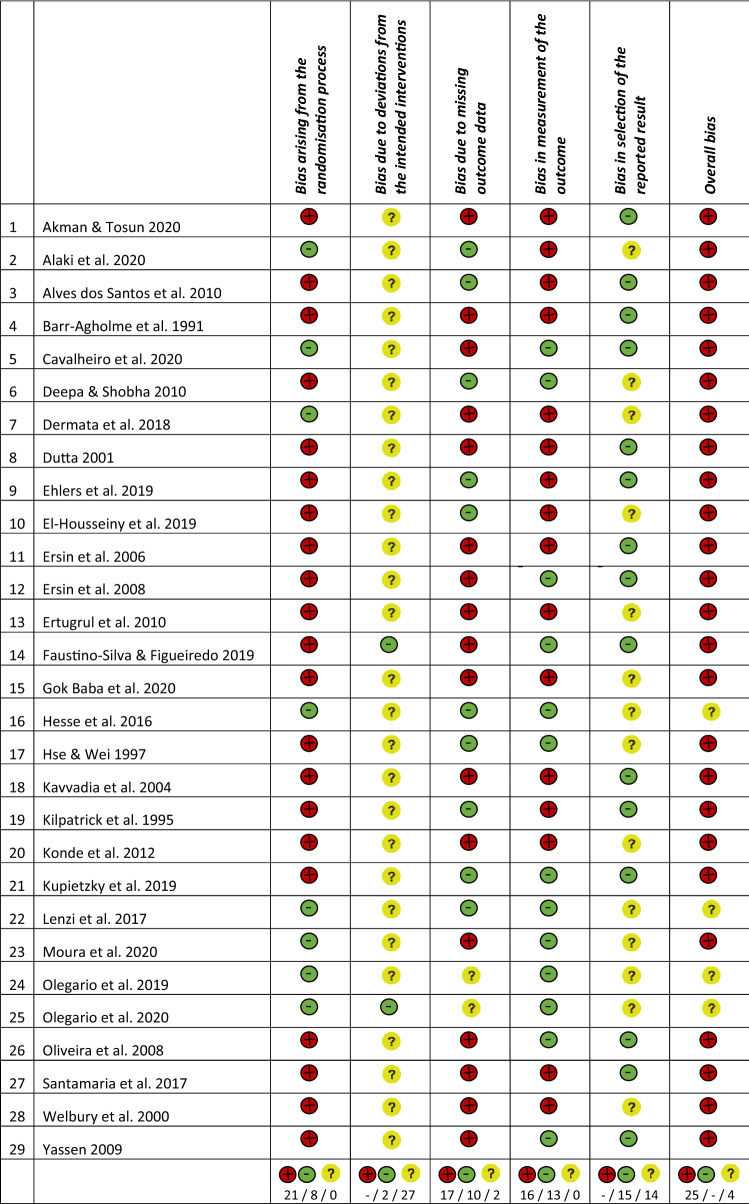
Quality assessment for the potential risk of bias in included studies

**Table 3:** RCTs with at least 1-year follow-up evaluating restorations on primary teeth: Study design, country, clinical setting, funding, restorative material under investigation, and inclusion criteria of included studies

Author, year	Study design	Country	Clinical setting (No of operators/evaluators) Funding (Yes (Source)/No)	Restorative material tested (material name)	Inclusion criteria (according to the authors)
Akman & Tosun, (2020)	RCT, split-mouth	Turkey	University (1/2) NR	CR (Filtek Z250) vs. two BFCR (Sonicfill, X-tra fil) vs. HVGIC (Equia)	Symptomless and vital 1^st^ or 2^nd^ primary molars ICDAS II code 4–5 proximal carious lesions At least 4 Class-II restorations/patient Each jaw with at least 2 proximal carious lesions in contact with adjacent teeth and in occlusion with the antagonist Radiolucency in outer 1/2 of dentine
Alaki et al., (2020)	RCT, parallel group	Saudi Arabia	University (1/1) Yes (University)	ZC (NR) vs. CSC (Z100)	Healthy 4–6-year-old children with opposed anterior teeth No systemic illness or dental developmental anomalies Caries susceptibility or selection of restorative materials to the best of current knowledge Minimally 2 carious surfaces ECC as defined by AAPD (2016) Cooperative patients (Frankl behaviour classification scale “positive” or “definitely positive”)
Alves dos Santos et al., (2010)	RCT, split-mouth	Brazil	University (2/2) NR	RMGIC (Vitremer) vs. C (Freedom) vs. CR (TPH Spectrum)	Mentally and physically healthy At least 2 occlusal (O) and/or occluso-proximal (MO or DO) primary caries lesions on primary molars No clinical or radiographic signs of pulpal/periradicular pathology and pathological wear All primary molars with occlusal and proximal contacts
Barr-Agholme et al., (1991)	RCT, split-mouth	Sweden	University (2/2) NR	A (Dispers alloy) vs. CR (P30)	NR
Cavalheiro et al., (2020)	RCT, parallel group	Brazil	University (1/1) Yes (CAPES)	BFCR (Filtek Bulk Fill) 15 ‘‘ vs. 7 ‘‘ dentine etching	NR
Deepa and Shoba, (2010)	RCT, split-mouth	India	University (1/1) NR	ART: Two HVGICs (Fuji IX, Amalgomer CR)	Bilateral pairs of caries Adjacent and opposing caries free teeth Opening > 0.9 mm
Dermata et al., (2018)	RCT, parallel group	Greece	University (6/4) NR	RMGIC (Vitremer) vs. CR (Filtek Z250)	Healthy (ASA I, II) Cooperative (Frankl 3, 4) children At least 1 1^st^ or 2^nd^ primary molar requiring a Class-II restoration
Dutta et al., (2001)	RCT, parallel group	India	NR (1/NR) NR	A (Solila) vs. RMGIC (Fuji II LC)	2–6 proximal cavities on primary molars
Ehlers et al., (2019)	RCT, split-mouth	Germany	Private dental practice (1/2) NR	Flowable BFCR (Venus Bulk-Fill) vs. C (Dyract eXtra)	At least 1 similar pair of carious primary molars in need of Class-II restorations
El-Housseiny et al., (2019)	RCT, split-mouth	Saudi Arabia	University (1/2) Yes (University)	GC (GCP Glass Fill) vs. RMGIC (Fuji II LC) vs. CR (Filtek Z250)	1 proximal lesion in at least 1 primary molar Radiographic evidence of caries extending to the inner half of enamel but not exceeding the inner half of dentine Proximal contact with adjacent teeth Occlusal contact with opposing teeth No indication for pulp therapy or other restorative treatment Predicted survival of 2 years until exfoliation
Ersin et al., (2006)	RCT, split-mouth	Turkey	School setting (3/2) Yes (GC Europe)	ART: CR (SureFil) vs. HVGIC (Fuji IX GP)	Dentinal lesions with an opening wide enough for the smallest excavator to enter
Ersin et al., (2008)	RCT, split-mouth	Turkey	School setting (3/2) Yes (3 M)	ART: One HVGIC (Ketac Molar) with and without Chlorhexidine containing cavity disinfectant	Bilateral matched pairs of carious primary molars Class-I or -II carious lesions extending into dentine Entrance large enough to allow access by the smallest excavator No evidence of pulpal involvement
Ertugrul et al., (2010)	RCT, split-mouth	Turkey	NR (3/1) NR	Conventional C (Compoglass F) vs. coloured C (Twinky Star)	At least 2 proximal carious lesions in primary molars Proximal contacts with adjacent healthy teeth No undermining of cusps by caries No caries lesions extending below the gingival margin of the cavity preparation Expected exfoliation time exceeding 2 years
Faustino-Silva & Figueiredo, (2019)	RCT, split-mouth	Brazil	University (1/1) Yes (University)	ART: Two HVGICs (Vitro Molar, Ketac Molar)	At least 1 primary molar in each of the dental quadrants Active cavitated carious lesions (depth: shallow-medium) Only occlusal surface involved
Gok Baba et al., (2021)	RCT, split-mouth	Turkey	University (1/1) NR	C (Dyract XP) vs. two HVGICS (Equia Forte, Chemfil Rock)	Frankl behaviour scale score 3–4 No history of allergy to any drugs/restorative materials Regular attendance at follow-up sessions 1^st^ and 2^nd^ primary molar with occlusal and proximal carious lesion No clinical symptoms requiring pulp therapy Neighbouring teeth present No occlusion problems Radiographically verified proximal dentine caries (carious lesion not in the pulpal 1/3 of dentine) Fanning’s root resorption level scale score Res_i_ or Res_1/4_ Normal monitoring of lamina dura Permanent successor present in normal position
Hesse et al., (2016)	RCT, parallel group	Brazil	School setting (4/1) Yes (Government grant)	ART: HVGIC (Fuji IX) with two different insertion techniques and two different surface protection materials	Cooperative behaviour At least one dentinal proximal caries lesion in a primary molar Maximum cavity dimensions: 2.0 mm mesio-distal, 2.5 mm bucco-lingual, and occluso-cervical No fistula or abscess near the selected tooth No pathological mobility of the selected tooth
Hse & Wei, (1997)	RCT, split-mouth	China	University (1/2) NR	C (Dyract) vs. CR (Prisma TPH)	Bilateral matched pairs of symptomless, unexposed, vital teeth
Kavvadia et al., (2004)	RCT, split-mouth	Greece	Private practice (2/2) Yes (3 M)	A (Dispersalloy) vs. C (F2000)	Vital teeth Proximal caries extending into dentine but not involving the pulp
Kilpatrick et al., (1995)	RCT, split-mouth	United Kingdom	NR (1/1)	GIC (Ketac Fil) vs. MRGIC (Ketac Silver)	Written informed consent At least 2 new proximal cavities in primary molars
Konde et al., (2012)	RCT, split mouth	India	University (NR/NR) NR	ART: HVGIC, Nanoionomer GIC (Fuji IX, Ketac Nano 100)	Single surface caries Comparable width and depth
Kupietzky et al., (2019)	RCT, parallel group	Israel	Private practice (1/2) NR	HVGIC (Equia) vs. CR (Filtek P60)	NR
Lenzi et al., (2017)	RCT, parallel group	Brazil	University (1/1) Yes (CAPES and FAPERGS)	CR (Filtek Z250) with ER vs. SE	Healthy and cooperative children At least 1 primary molar with moderately deep dentine caries No sensitivity, spontaneous pain, swelling, fistula, mobility No periapical/ interradicular radiolucency or other signs of pulp necrosis
Moura et al., (2020)	RCT, parallel group	Brazil	School setting (2/1) NR	ART: Two GICs (Ketac Molar, Vitro Molar)	Presence of dentine caries without painful symptomatology or pulp involvement
Olegario et al., (2019)	RCT, parallel group	Brazil	School setting (4/1) NR	ART: GIC (Equia Fil) vs. C (Dyract) vs. GC (CAR-glass)	Good behaviour and health Possibility of following-up at least 1 year Accepted consent form Minimum 1 primary molar with occlusal dentine caries accessible to hand instruments
Olegario et al., (2020)	RCT, parallel group	Brazil	School setting (2/1) NR	ART: Three HVGICs (Fuji IX Gold Label, Vitro Molar, Maxxion R)	Good behaviour and health Possibility of following-up at least 1 year Accepted consent form Minimum 1 primary molar with occlusal dentine caries accessible to hand instruments Absence of fistula or abscess No pain, pulp exposure, mobility
Oliveira et al., (2008)	RCT, split-mouth	Brazil	University (1/2) NR	CR (TPH Spectrum) with bevelled vs. no bevelled margin	Mentally and physically healthy At least 2 Class-I lesions on primary molars No indication for periodontal or pulp therapy
Santamaria et al., (2017)	RCT, parallel group	Germany	University (12/2) Yes (University)	NRCT vs. HT (PMC) vs. C (Dyract)	Primary molar with 2-surface occluso-proximal dentine caries (ICDAS coded 3–5) No clinical or radiographic symptoms of pulpal/periradicular pathology No systemic disease Willingness to participate
Welbury et al., (1991)	RCT, split mouth (partially)	UK	University (2/2) Yes (Scholarship)	A (Amalcap) vs. GIC (Ketac Fil)	Cavity on previously undamaged surface or cavity due to secondary caries or restoration loss
Yassen, (2009)	RCT, split-mouth	Iraq	School setting (1/1) NR	ART: One GIC (Ionofil) with and without cavity conditioner	Matched pair of occlusal caries of similar size extending into dentine with an entrance wide enough for hand instruments

*RCT* randomised controlled trial, *A* amalgam, *AAPD* American Academy of Pediatric Dentistry, *ART* Atraumatic restorative treatment, *BFCR* bulk-fill composite resin, *C* compomer, *CR* composite resin, *CSC* composite strip crown, *ECC* early childhood caries, *ER* etch and rinse, *F* fracture, *GC* glass carbomer, *GIC* glass-ionomer cement, *HT* Hall technique, *HVGIC* high viscosity glass-ionomer cement, *ICDAS* International Caries Detection and Assessment System,  *MRGIC* metal-reinforced glass-ionomer cement, *NR* not reported, *NRCT* non-restorative caries treatment, *RMGIC* resin-modified glass-ionomer cement, *SE* self-etch, *PMC* preformed metal crown, *ZC* zirconia crown

### Quality assessment of the included studies

**Fig. 2:**
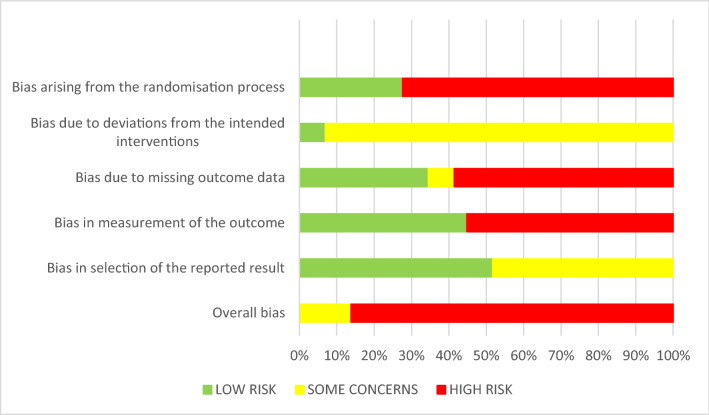
Quality assessment for the potential risk of bias

Twenty-five included RCTs were of an overall high risk of bias. For the remaining 4 studies, the overall risk of bias was unclear. The main reasons for that were: the randomisation process (69% of the trials), missing outcome data (59%) or measurement of the outcome (52%) (Fig. 2 and Table 2). Most of the studies that were marked as high risk due to the randomisation process, had no information on allocation concealment.

### Bias arising from the randomisation process

Twenty-one studies were rated as having a high risk of selection bias based on inadequate information provided about the randomisation process and/or the allocation sequence concealment. The remaining 8 studies were of low risk of bias (Alaki et al. 2020; Cavalheiro et al. 2020; Dermata et al. 2018; Hesse et al. 2016; Lenzi et al. 2017; Moura et al. 2020; Olegario et al. 2019, 2020). A computer-generated randomisation was applied in several studies (Alaki et al. 2020; Cavalheiro et al. 2020; Dermata et al. 2018; Lenzi et al. 2017; Olegario et al. 2019, 2020). Opaque, sealed envelopes (Alaki et al. 2020; Cavalheiro et al. 2020; Moura et al. 2020; Olegario et al. 2020) or a central allocation (Dermata et al. 2018; Lenzi et al. 2017; Olegario et al. 2019) were used to conceal the randomisation sequence.

### Bias due to deviations from the intended interventions

In many studies, blinding of participants and trial personnel appealed to be not feasible due to the different materials used for the restoration of primary teeth. Two studies (Faustino-Silva and Figueiredo 2019; Olegario et al. 2020) were graded as low risk of bias. For the other 27 studies, some concerns persisted regarding performance bias, and therefore, these studies were rated with an unclear risk.

### Bias due to missing outcome data

Seventeen studies exhibited a high risk of bias for missing outcome data (58.6%) because of drop-out rates exceeding 10%. In 2 studies, there were some concerns about bias due to missing outcome data (Olegario et al. 2019, 2020). Given the drop-out rates of < 10% in the remaining 10 studies, they were judged as having a low risk of attrition bias (Alaki et al. 2020; Alves dos Santos et al. 2010; Deepa and Shobha 2010; Ehlers et al. 2019; El-Housseiny et al. 2019; Hesse et al. 2016; Hse and Wei 1997; Kilpatrick et al. 1995; Kupietzky et al. 2019; Lenzi et al. 2017).

### Bias in measurement of the outcome

Given the fact that several included studies compared the clinical performance of different restorative materials or that operator and outcome assessor were the same person, 55.2% (n = 16) of studies were rated as being of high risk of detection bias. In 44.8% (n = 13) of published studies, the outcome assessors were adequately blinded to the intervention.

### Bias in selection of the reported results

None of the studies were at high risk of bias in selection of the reported results. In 48.3% (n = 14) of studies the risk of bias was rated as “unclear” because of incomplete reporting of results for pre-specified outcome criteria (Alaki et al. 2020; Deepa and Shobha 2010; Dermata et al. 2018; El-Housseiny et al. 2019; Ertugrul et al. 2010; Gok Baba et al. 2021; Hesse et al. 2016; Hse and Wei 1997; Konde et al. 2012; Lenzi et al. 2017; Moura et al. 2020; Olegario et al. 2019, 2020; Welbury et al. 1991). The rest of the included studies reported the results completely (51.7%, n = 15).

## Reported outcomes for the restorative materials

### Amalgam

Tables 3 and 4 show the reported outcomes for the restorative materials including four trials that investigated the use of amalgam in primary teeth (Barr-Agholme et al. 1991; Dutta et al. 2001; Kavvadia et al. 2004; Welbury et al. 1991). The studies compared amalgam with composite resin (Barr-Agholme et al. 1991), compomer (Kavvadia et al. 2004), GIC (Welbury et al. 1991) and RMGIC (Dutta et al. 2001). The four studies used the USPHS criteria or Ryge’s criteria to evaluate clinical performance. The follow-up period ranged from 1 to 5 years. There was a high variation of annual failure rates for amalgam ranging from 1 to 28%. The study by Kavvadia et al. (2004) was the only one using rubber dam and reported the lowest AFR of 1% (Kavvadia et al. 2004). Three studies reported on secondary caries ranging from 9 to 12.5%, two studies on marginal adaptation ranging from 17 to 32%, and one study on loss of retention of 2–3.4%.

Given the overall high risk of bias among the included studies, the results for amalgam restorations are based on a low quality of evidence.

### Glass-ionomer cements

The clinical performance of conventional (GIC), metal-reinforced (MRGIC), resin-modified (RMGIC), or high-viscosity glass-ionomer cements (HVGIC) was assessed in primary dentition in 9 included randomised controlled clinical trials (Akman and Tosun 2020; Alves dos Santos et al. 2010; Dermata et al. 2018; Dutta et al. 2001; El-Housseiny et al. 2019; Gok Baba et al. 2021; Kilpatrick et al. 1995; Kupietzky et al. 2019; Welbury et al. 1991). The comparators chosen were amalgam (Dutta et al. 2001; Welbury et al. 1991), compomer (Alves dos Santos et al. 2010; Gok Baba et al. 2021), composite resin (Akman and Tosun 2020; Alves dos Santos et al. 2010; Dermata et al. 2018; El-Housseiny et al. 2019; Kupietzky et al. 2019), bulk-fill composite resin (Akman and Tosun 2020), another type of glass-ionomer cement (Gok Baba et al. 2021; Kilpatrick et al. 1995), and glass carbomer (El-Housseiny et al. 2019). One trial solely compared glass-ionomer cements (Kilpatrick et al. 1995). If reported, isolation was achieved by use of cotton rolls in 3 studies (Akman and Tosun 2020; Gok Baba et al. 2021; Welbury et al. 1991) or by rubber dam in 4 studies (Alves dos Santos et al. 2010; Dermata et al. 2018; El-Housseiny et al. 2019; Kupietzky et al. 2019). The follow-up of included studies ranged from 1 to 5 years. Most commonly modified USPHS criteria (8 trials) were used to evaluate the clinical performance. Kupietzky et al. (2019) described their own clinical and radiographic evaluation criteria and assessed the clinical performance by means of photographs and radiographs (Kupietzky et al. 2019). The annual failure rates were 7.6–16.6% for GIC, 29.9% for MRGIC, 1.9–16.9% for RMGIC, and 2.9–25.6% for HVGIC. Reported reasons for failure of glass-ionomer cements were loss of retention (2% (per protocol analysis) up to 25.6%), fractures (0–15.2%), marginal adaptation (0–13.5%), and secondary caries (0–7%). In comparison to amalgam, GIC exhibited a higher (Welbury et al. 1991) and RMGIC a lower failure rate (Dutta et al. 2001). RMGIC achieved similar or slightly higher failure rates compared to compomer and composite resin restorations (Alves dos Santos et al. 2010; Dermata et al. 2018; El-Housseiny et al. 2019). The results for HVGIC showed a high variation. Whereas Akman & Tosun (2020) reported an acceptable clinical performance of HVGIC for Class-II restorations after 1 year (Akman and Tosun 2020), Class-II restorations with compomer and composite resin outperformed HVGIC in two other trials after 1–3 years (Gok Baba et al. 2021; Kupietzky et al. 2019). MRGIC presented the highest failure rate in Class-II restorations with loss of anatomical form and marginal integrity over time (Kilpatrick et al. 1995).

All studies are at high risk of bias, which results in a low quality of evidence for these findings on the restorative treatment of carious primary teeth with various glass-ionomer cements.

### Glass carbomer

Only one included clinical study (El-Housseiny et al. 2019) reported on glass carbomer restorations after drilling in primary teeth. Failure rates of glass carbomer were extremely high (up to 46.2% AFR) compared to composite resin (2.1% AFR) or RMGIC (up to 7.5% AFR). Main reasons for failure were marginal adaptation and anatomical form (fracture).

The findings from the single study evaluating the use of glass carbomer in Class-II cavities of primary molars are based on evidence of low quality.

### Compomer

Seven of the included randomised controlled clinical trials investigated the clinical effectiveness of compomer restorations in primary teeth (Alves dos Santos et al. 2010; Ehlers et al. 2019; Ertugrul et al. 2010; Gok Baba et al. 2021; Hse and Wei 1997; Kavvadia et al. 2004; Santamaria et al. 2017). The following restorative materials were selected as comparators in these trials: amalgam (Kavvadia et al. 2004), RMGIC (Alves dos Santos et al. 2010), HVGIC (Gok Baba et al. 2021), another compomer (Ertugrul et al. 2010), composite resin (Alves dos Santos et al. 2010; Hse and Wei 1997), bulk-fill composite resin (Ehlers et al. 2019), and alternative caries management approaches (Santamaria et al. 2017). In the majority of trials, Class-II restorations were examined. Rubber dam was used to isolate the teeth during restorative treatment in 3 trials (Alves dos Santos et al. 2010; Hse and Wei 1997; Kavvadia et al. 2004) and cotton rolls with saliva ejector were chosen in another 2 trials (Ertugrul et al. 2010; Gok Baba et al. 2021). Again, the clinical performance of restorations was most commonly assessed by modified USPHS criteria (5 trials). In one trial, FDI criteria were chosen (Ehlers et al. 2019). In another trial, the categories “success”, “minor failure” and “major failure” were described for outcome assessment (Santamaria et al. 2017). The annual failure rate of compomer restorations varied from 0 to 14.7%. In primary teeth, compomer restorations exhibited a similar clinical performance compared to amalgam (Kavvadia et al. 2004), RMGIC and composite resin (Alves dos Santos et al. 2010), composite resin (Hse and Wei 1997), and a flowable bulk-fill composite resin applied without cover layer (Ehlers et al. 2019). In comparison to two HVGICs, the compomer achieved more favourable retention rates (AFR 3.6% vs. 13.4–25.6%) (Gok Baba et al. 2021). One trial including a tooth coloured and a coloured compomer found comparable survival rates after 1 year for both materials in Class-II restorations (Ertugrul et al. 2010). Overall, restorations failed due to secondary caries (0–15%) and loss of retention (1.7–3.6%). One trial reported a combination of fracture and loss of retention in 9% of cases (Santamaria et al. 2017).

The results for compomers are based on studies with high risk of bias leading to a low quality of evidence.

### Composite resin

Clinical performance of composite resins in primary teeth was reported in 11 of the included studies, compared to compomer in 3 studies (Alves dos Santos et al. 2010; Ehlers et al. 2019; Hse and Wei 1997), to GIC or RMGIC in 5 studies (Akman and Tosun 2020; Alves dos Santos et al. 2010; Dermata et al. 2018; El-Housseiny et al. 2019; Kupietzky et al. 2019), to amalgam in 1 study (Barr-Agholme et al. 1991), and to glass carbomer in another study (El-Housseiny et al. 2019). Three studies included only composite resins in their experimental groups, testing parameters of the application technique. Therefore, the variables were: (i) dentine etching time 15 s vs. 7 s, (ii) etch-and-rinse vs. self-etch adhesion, (iii) bevel vs. no bevel during cavity preparation (Cavalheiro et al. 2020; Lenzi et al. 2017; Oliveira et al. 2008). Most of the studies used hybrid composite resin and 3 studies reported results for bulk-fill composite resins (Akman and Tosun 2020; Cavalheiro et al. 2020; Ehlers et al. 2019). The most commonly used evaluation criteria were the modified USPHS criteria and duration of the studies was 1–4 years.

Hybrid composite resin restorations in primary teeth exhibited 0–19.5% AFR, while bulk-fill composite resins showed 0–16.9% AFR. Two separate studies reported 0% failure rate for bulk-fill composite resins with different evaluation criteria (FDI and modified USPHS) (Cavalheiro et al. 2020; Ehlers et al. 2019). Compared to amalgam restorations, amalgam demonstrated higher failure rates and more failures due to inferior marginal adaptation (Barr-Agholme et al. 1991). Similar or lower failure rates were recorded for composite resins compared to GIC/RMGIC, except from one study showing lower failure rates for RMGIC (El-Housseiny et al. 2019). Same or higher failure rates were shown for composite resins compared to compomers.

Etching of dentine for 15 s in comparison to 7 s resulted in comparable survival rates of bulk-fill composite resin restorations. Generally speaking, the clinical outcome was more favourable when phosphoric acid etching of dentine was performed for 7 s in contrast to 15 s (Cavalheiro et al. 2020). Etch-and-rinse adhesive technique demonstrated higher failure rates (27.8%) compared to self-etch (10.3%) after 1.5 years (Lenzi et al. 2017). When no bevel was placed, failure rate was twice as high of a value compared to bevel placement and twice as many lost teeth due to secondary caries (Oliveira et al. 2008).

The results for composite resin restorations in primary teeth are mainly based on studies with a high risk of bias (10/11) and 1 study with an unclear risk of bias (1/11). Therefore, the evidence is of low to unclear quality.

### New biomaterials

As far as new restorative materials with bio-active molecules are concerned (Imazato et al. 2014), the search retrieved no results for RCTs on the clinical effectiveness of these restorative materials for the treatment of carious primary teeth.

### Crowns

Two studies on crowns were included in the systematic review. Both studies were conducted prospectively in dental school settings (Alaki et al. 2020; Santamaria et al. 2017). They were carried out as randomised trials (parallel group design) comparing different restorative materials or techniques. In one study, zirconia crowns (ZC) and composite strip crowns (CSC) filled with a hybrid composite with fine particles (Z100) were evaluated (Alaki et al. 2020). The other study compared preformed metal crowns (PMC) placed using the Hall-technique with compomer restorations and non-restorative caries treatment (Santamaria et al. 2017). The follow-up times varied from 1 to 2.5 years with 40 to 60 restorations being followed. For evaluation, one study used the USPHS criteria (Alaki et al. 2020) and in the other one own criteria (successful, minor and major failures) were applied (Santamaria et al. 2017). The annual failure rate ranged between 3.3% (ZC) and 15% (CSC). Reasons for failure were secondary caries (2.5–6.7%) and loss or fracture of the restorations (2.5–38.3%).

Again, there is a low quality of evidence as the findings are based on studies with a high risk of bias.

### Class-I vs. Class-II restorations

Three of the four trials solely evaluated the clinical performance of amalgam in Class-II cavities (Barr-Agholme et al. 1991; Dutta et al. 2001; Kavvadia et al. 2004) however, no comparison with Class-I restorations was made in the same study under the same standardised parameters. In the ART studies that compared Class-I restorations with Class-II, there was a higher annual failure rate for Class-II restorations (5–28.9%) compared to 1.2–14.7% for Class-I. Two out of nine studies with glass-ionomer cements included both Class-I and Class-II restorations (Alves dos Santos et al. 2010; Welbury et al. 1991) and the remaining examined only Class-II restorations. Higher survival rates were shown in Class-I restorations than in Class-II in 4 years (Alves dos Santos et al. 2010; Welbury et al. 1991); however, Welbury et al. 1991 did not provide separate data on the two types of restorations (Welbury et al. 1991). Two trials assessed Class-II compomer restorations combined with additional cavity classes and composite resin restorations compared with other cavity classes, namely, Class-I or Class-III and Class-V cavities (Alves dos Santos et al. 2010; Hse and Wei 1997). Again, Alves dos Santos et al. 2010 reported higher survival rates in Class-I compomer and composite resin restorations than in Class-II in 4 years (Alves dos Santos et al. 2010; Welbury et al. 1991). Hse and Wei 1997 did not give separate data on Class-II, -III and -V restorations but concluded that compomers can be suitable for restoring Class-III and Class-V cavities as well and not only Class-II (Alves dos Santos et al. 2010; Hse and Wei 1997). In addition, six studies reported on Class-II composite resin restorations, one on Class-I and 2 studies gave no information on cavity size. As far as crowns are concerned, 1 study included anterior primary teeth with at least two surfaces affected by carious lesions without mentioning the lesion depth (Alaki et al. 2020). The other RCT included primary molars with Class-II carious lesions extending into the dentine (Santamaria et al. 2017). Further comparison of the results from different study settings was not possible.

### Restorative materials used for atraumatic restorative treatment (ART)

Ten trials investigated the use of Atraumatic Restorative Treatment (ART) in primary teeth (Deepa and Shobha 2010; Ersin et al. 2008, 2006; Faustino-Silva and Figueiredo 2019; Hesse et al. 2016; Konde et al. 2012; Moura et al. 2020; Olegario et al. 2019, 2020; Yassen 2009). Five studies evaluated the clinical performance of various types of GIC (conventional GIC, RMGIC, HVGIC, and glass carbomer) for ART (Deepa and Shobha 2010; Faustino-Silva and Figueiredo 2019; Konde et al. 2012; Moura et al. 2020; Olegario et al. 2020) and three studies evaluated different application techniques of GIC for ART (Ersin et al. 2008; Hesse et al. 2016; Yassen 2009). In one study, a compomer was compared with a GIC and a glass carbomer (Olegario et al. 2019), and in another a GIC with a composite resin (Ersin et al. 2006). Eight studies used cotton rolls as a form of isolation, and in the remaining 2 studies the isolation technique was not reported (Hesse et al. 2016; Konde et al. 2012). Various criteria were used to evaluate clinical performance and included USPHS criteria (Ersin et al. 2008, 2006; Faustino-Silva and Figueiredo 2019; Konde et al. 2012), special ART criteria or Frencken’s (Deepa and Shobha 2010; Faustino-Silva and Figueiredo 2019; Hesse et al. 2016; Moura et al. 2020; Yassen 2009). Two studies used Roeleveld criteria (Olegario et al. 2019, 2020). The follow-up period ranged from 1 to 4 years. There was a high variation of the AFR ranging from 1.2 to 37.1%. Only one study reported secondary caries as a reason for failure (Konde et al. 2012), due to the nature of ART, where caries is inherently left below the restoration at placement. The use of polyacrylic acid cavity conditioner prior to placement of GIC did not improve the success rate of the restoration (Yassen 2009). Using a bilayer technique for placement of HVGIC in proximal lesions positively influenced the survival rate when compared to conventional one step placement (Hesse et al. 2016). In the same study, the use of a nano-filled coating increased the restoration longevity. The use of a cavity disinfectant prior to GIC placement did not affect the success rate for both Class-I and Class-II ART restorations (Ersin et al. 2008). The use of composite resin in one study did not show an improved survival rate, when compared to HVGIC (Ersin et al. 2006). Fracture of restoration or loss of retention were the most common causes of failure (2.6–54% for fracture and 5.2–44.7% for loss of retention).

All these findings are premised on studies with a high or unclear risk of bias resulting in a low of unclear quality of evidence for restorative materials chosen for ART in primary dentition.

### Quantitative synthesis of the included studies

The lack of standardised protocols impeded a valid interpretation of the actual results through pooled estimates. Substantial differences in the implemented interventions, follow-up duration and investigated outcomes indicated significant heterogeneity. Moreover, and probably more notably, the risk of bias assessment of the included studies indicated high risk in all, except in 4 studies, that were evaluated at unclear risk (rated with 'Some concerns') (Table 2). Again, significant clinical and methodological heterogeneity among these 4 studies was identified. Therefore, a meta-analysis was not feasible.

**Table 4:** RCTs with at least 1-year follow-up evaluating restorations on primary teeth: the systematic review results for the restorative treatment

Author, year	Restorative material (material name)	Restoration class Isolation	Follow-up (years)	Included participants in total [age (years)] Gender (male:female)	Restoration at baseline/Last follow-up per group	Evaluation criteria	Failure rate /Annual failure rate/Annual failure rate according to Opdam et al., 2014 (Opdam et al. 2014)	**Reasons for failure** secondary caries (SC) Marginal adaptation/integrity (MA/MI) Fractures (F) Loss of Retention (LR)
Akman & Tosun, (2020)	HVGIC (Equia Fil) CR: Z250 BFCR^1^: SonicFill BFCR^2^: X-tra fil	Class II Cotton rolls, saliva ejector	1	30 (6–10) NR	HVGIC: 40/34 CR: 40/32 BFCR^1^: 40/34 BFCR^2^: 40/34	Modified USPHS	**HVGIC:** 2.9%/2.9%/2.9%§ **CR:** 0%/0%/0%§ **BFCR**^**1**^**:** 0%/0%/0%§ **BFCR**^**2**^**:** 0%/0%/0%§	**HVGIC:** SC: 0%* MA: 0%* F: 0%* LR: 2.9%* **CR, BFCR**^**1**^**, BFCR**^**2**^**:** SC: 0%* MA: 0%* F: 0%* LR: 0%*
Alaki et al., (2020)	Zirconia crown (NR) Strip crown (NR)	NR	1	32 (3–5.5) 12:20	ZC: 60/60 CSC: 60/60	USPHS Silness and Löe Smith and Knight Tooth Wear Index	**ZC:** 3.3%/3.3%/3.3% **CSC:** 15%**/15%**/15%**	**ZC:** SC: 0% MI: 0% F: 0% LR: 3.3% **CSC:** SC: 6.7% MI: 0% F: 0% LR: 38.3%
Alves dos Santos et al., (2010)	C (Freedom) RMGIC (Vitremer) CR (TPH Spectrum)	Class I Class II Rubber dam	4	48 (3–9) NR	C: 51/43 RMGIC: 46/34 CR: 44/33	Modified USPHS	**C:** 25.8%*/1.68%*† 15.7%/3.9%/4.2% **RMGIC:** 38.7%*/1.86%*† 26.1%/6.5%/7.3% **CR:** 35.5%*/1.82%*† 25%/6.3%/6.9%	**C:** Class-I MA/marginal discolouration/SC: 3.9% Class-II SC: 11.8% **RMGIC:** Class-I F: 15.2% Class-II SC, LR: 10.9% **CR:** Class-I MA: 11.4% Class-II MA, SC: 13.6%
Barr-Agholme et al., (1991)	A (Dispersalloy) CR (P30)	Class II Cotton rolls	2	43 (4–8) 25:18	A: 64/52 CR: 55/34	USPHS	**A:** 32%*/16%/17.5% **CR:** 12%*/6%/6.2%	**A:** SC: 9%* MA: 32%* F: NR LR: NR **CR:** SC: 6%* MA: 6%* F: NR LR: NR
Cavalheiro et al., (2020)	BFCR (Filtek Bulk Fill)	NR Rubber dam	1.5	62 (5–8) 24:38	BFCR 15’’: 50/43 BFCR 7’’: 50/45	FDI	**BFCR 15’’:** 24.3%*/16.9%*/16.9%† **BFCR 7’’:** 8.6%*/5.7%*/5.8%†	**BFCR 15’’:** SC: 2%* MA: 8%* F: 6%* LR: NR **BFCR 7’’:** SC: 2%* MA: 4%* F: 2%* LR: NR
Deepa and Shobha, (2010)	HVGIC^1^ (Fuji IX) HVGIC^2^ (Amalgomer CR)	Class I Class II Cotton rolls	1	100 (4–9) NR	HVGIC^1^: 39/39 Class I 61/61 Class II HVGIC^2^: 39/39 Class I 61/61 Class II	Frencken’s	**HVGIC**^**1**^**:** Cumulative 9%*/9%/9% Class I 5%*/5%/5% Class II 11.5%*/11.5%/11.5% **HVGIC**^**2**^**:** Cumulative 4%*/4%/4% Class I 3%*/3%/3% Class II 5%*/5%/5%	NR
Dermata et al., (2018)	RMGIC (Vitremer) CR (Filtek Z250)	Class II Rubber dam	2	55 (4–7.5) 24:31	RMGIC: 63/55 CR: 61/49	Modified USPHS	**RMGIC:** 16%*/8%/8.4%‡ **CR:** 16%*/8%/8.4%‡	**RMGIC:** SC: 7%*‡ MA: 4%*‡ F: NR LR: 2%*‡ **CR:** SC: 13%*‡ MA: 2%*‡ F: NR LR: 6%*‡
Dutta et al., (2001)	A (Solila) RMGIC (Fuji II LC)	Class II NR	1	120 (4–9) NR	A: 120/100 RMGIC: 360/290	Modified USPHS	**A:** 28%*/28%/28% **RMGIC:** 16.9%*/16.9%/16.9%	**A:** SC: 12.5%* MA: 17%* F: 0%* LR: NR **RMGIC:** SC: 4.7% * MA: 13.5%* F: 1.2%* LR: NR
Ehlers et al., (2019)	C (Dyract eXtra) BFCR (Venus Bulk-Fill)	Class II NR	1	32 (4–9) 17:15	C: 50/50 BFCR: 50/49	FDI	**C:** 0%*/0%/0% **BFCR:** 0%*/0%/0%	**C, BFCR:** SC: 0% MA: 0% F: 0% LR: 0%
El-Housseiny et al., (2019)	RMGIC (GC Fuji II LC) GC (GCP Glass Fill) CR (Filtek Z250)	Class II Rubber dam	1	50 (4–8) NR	RMGIC: 54/53 GC: 54/52 CR: 54/47	Cvar and Ryge	**RMGIC:** 1.9–7.5%*/1.9–7.5%/1.9–7.5% **GC:** 5.8–46.2%*/5.8–46.2%/5.8–46.2% **CR:** 2.1%*/2.1%/2.1%	**RMGIC:** SC: 0% MA: 7.5% F: 0% LR: NR **GC:** SC: 0% MA: 46.2% F: 25.0% LR: NR **CR:** SC: 0% MA: 2.1% F: 0% LR: NR
Ersin et al., (2006)	HVGIC (Fuji IX GP) CR (Surefil)	Class I Class II Cotton rolls	2	219 (6–10) NR	HVGIC: 119/106 Class I 96/70 Class II CR: 111/95 Class I 93/73 Class II	Modified USPHS	**HVGIC:** Cumulative 11.5%/5.8%/5.9% Class I 3.3*%/1.6%/1.7%† Class II 23.9*%/12%/12.8%† **CR:** Cumulative 12.9%/6.5%/6.7% Class I 9%*/4.5%/4.6%† Class II 18%*/9%/9.5%†	**HVGIC:** Class I SC: NR MA: 2.7%* F: 2.8%* LR: NR Class II SC: NR MA: 24.6%* F: 24.6%* LR: NR **CR:** Class I SC: NR MA: 2.3%* F: 2.7%* LR: NR Class II SC: NR MA: 19.3%* F: 19.3%* LR: NR
Ersin et al., (2008)	HVGIC (Ketac Molar) without disinfectant HVGIC (Ketac Molar) with Concepsis (2% chlorhexidine–gluconate-based disinfectant)	Class I Class II Cotton rolls	2	126 (6–8) 56:69, conflicting information	HVGIC (without disinfectant): 56/51 Class I 70/58 Class II HVGIC (with disinfectant): 53/46 Class I 70/47 Class II	Modified USPHS	**HVGIC without disinfectant:** Cumulative 17.4%/8.7%/9.1% Class I 2.3%*/1.2%/1.2%† Class II 30.6%*/15.3%/16.7%† **HVGIC with disinfectant:** Cumulative 21%/10.5%/11.1% Class I 4.8%*/2.4%/2.4%† Class II 36.1%*/18%/20.1%†	**HVGIC without disinfectant:** Class I SC: NR MA: 2.6% F: 2.6%* LR: NR Class II SC:NR MA: 34%* F: 34%* LR: NR **HVGIC with disinfectant:** Class I SC: NR MA: 2.6%* F: 5.3%* LR:NR Class II SC:NR MA:38%* F:38%* LR:NR
Ertugrul et al., (2010)	C^1^ (Compoglass F) C^2^ (Twinky Star)	Class II Cotton rolls, saliva ejector	1	98 (5–10) 54:44	C^1^: 98/86 C^2^: 98/85	Modified USPHS	**C**^**1**^**:** 4.3%*/4.3%/4.3%† **C**^**2**^**:** 7%*/7%/7%†	**C**^**1**^**:** SC: 1% MA: 0% F: 0% LR:NR **C**^**2**^**:** SC: 0% MA: 0% F: 0% LR:NR
Faustino-Silva & Figueiredo, (2019)	HVGIC^1^ (Vitro Molar) HVGIC^2^ (Ketac Molar Easymix)	Class I Cotton rolls	4	25 (1.5–3) 12:13	HVGIC^1^: 50/38 HVGIC^2^: 50/38	ART Criteria Modified USPHS	**HVGIC**^**1**^**:** 13.2%*/3.3%/3.5%† **HVGIC**^**2**^**:** 21.1%*/5.3%/5.8%†	**HVGIC**^**1**^**:** SC: 0%* MA: 7.9%* F:10.6%* LR: 5.2%* **HVGIC**^**2**^**:** SC: 0%* MA:13.2%* F:10.6%* LR:10.6%*
Gok Baba et al., (2021)	C (Dyract XP) HVGIC^1^ (Equia Forte) HVGIC^2^ (Chemfil Rock)	Class II Cotton rolls, saliva ejector	1	63 (4–7) 31:32	C: 83/83 HVGIC^1^: 86/86 HVGIC^2^: 82/82	Modified USPHS	**C:** 3.6%/3.6%/3.6% **HVGIC**^**1**^**:** 25.6%/25.6%/25.6% **HVGIC**^**2**^**:** 13.4%/13.4%/13.4%	**C:** SC: 0% MA: 0% F: 0% LR: 3.6% **HVGIC**^**1**^**:** SC: 0% MA: 0% F: 0% LR:25.6% **HVGIC**^**2**^**:** SC: 0% MA: 0% F: 0% LR:13.4%
Hesse et al., (2016)	HVGIC^1^: Conventional HVGIC (GC Fuji IX) insertion, coat (petroleum jelly) HVGIC^2^: Bilayer HVGIC technique, coat (petroleum jelly) HVGIC^3^: Conventional HVGIC insertion, coat (GC G-Coat Plus) HVGIC^4^: Bilayer HVGIC technique, coat (GC G-Coat Plus)	Class II NR	3	208 (6–7) 108:100	HVGIC^1^: 55/53 HVGIC^2^: 54/50 HVGIC^3^: 42/37 HVGIC^4^: 57/50	ART Criteria	**HVGIC**^**1**^**:** 69.1%*/23%/32.4%† **HVGIC**^**2**^**:** 35.8%*/11.9%/13.7%† **HVGIC**^**3**^**:** 46.3%*/15.4%/18.7%† **HVGIC**^**4**^**:** 35.1%*/11.7%/13.4%†	**HVGIC**^**1**^**:** SC: NR MA: NR F: 53% LR: NR **HVGIC**^**2**^**:** SC: NR MA: NR F: 16% LR: NR **HVGIC**^**3**^**:** SC: NR MA: NR F: 54% LR: NR **HVGIC**^**4**^**:** SC: NR MA: NR F: 24% LR: NR
Hse & Wei, (1997)	C (Dyract) CR (Prisma TPH)	Class I Class II Class V Rubber dam	1	36 (4–7) NR	C: 60/60 CR: 60/60	Modified USPHS	**C:** 1.7%*/1.7%/1.7% **CR:** 1.7%*/1.7%/1.7%	**C:** SC: 0% MA: 0% F: 0% LR: 1.7% **CR:** SC: 0% MA: 0% F: 0% LR: 1.7%
Kavvadia et al., (2004)	A (Dispersalloy) C (F2000)	Class II Rubber dam	2	75 (6–9) NR	A: 75/57 C: 75/57	Modified Ryge	**A:** 2%/1%/1% **C:** 2%/1%/1%	**A:** SC: NR MA: NR F: NR LR: 2%* **C:** SC: 2%* MA: NR F: NR LR: NR
Kilpatrick et al., (1995)	GIC (Ketac Fil) MRGIC (Ketac Silver)	Class II NR	1.5 (mean follow-up)	37 (4 years 10 mos-10 years 10 mos) 21:16	GIC: 46/46 MRGIC: 46/46	Modified USPHS	**GIC:** 23.9%*/15.9%/16.6%† **MRGIC:** 41.3%*/27.5%/29.9%†	**GIC:** SC: 4% MA:NR F:NR LR:NR **MRGIC:** SC: 9% MA:NR F:NR LR:NR
Konde et al., (2012)	RMGIC (Ketac Nano 100) HVGIC (Fuji IX)	Class I NR	1	50 (5–8) NR	RMGIC: 50/47 HVGIC: 50/47	USPHS	**RMGIC:** 2%/2%/2% **HVGIC:** 12.8%/12.8%/12.8%	**RMGIC:** SC: 2% MA: NR F: NR LR: NR **HVGIC:** SC: 12.8% MA: NR F: NR LR: NR
Kupietzky et al., (2019)	HVGIC (Equia) CR (Filtek P60)	Class II Rubber dam	3	87 (mean 6.7) 43:44	HVGIC: 70/70 CR: 61/61	Own: Clinical: retention, occlusal defects, contact point Radiographically: homogeneity of material, concavity defect of proximal wall, secondary caries, presence of radiolucent affected dentine	**HVGIC:** 17.1%*/5.7%/6.1%§ **CR:** 4.9%*/1.6%/1.7%§	**HVGIC:** SC: 0% MA: 7.1% F:NR LR: 17.1% **CR:** SC: 3.4% MA: 4.9% F:NR LR: 4.9%
Lenzi et al., (2017)	CR (Filtek Z250)	NR Rubber dam	1.5	44 (5–10) 20:24	ER: 45/40 SE: 45/40	Modified USPHS	**ER:** 27.8%/18.53%/19.5%† **SE:** 10.3%/6.87%/7.0%†	NR
Moura et al., (2020)	HVGIC (Ketac Molar) GIC (Vitro molar)	Class I Class II Class III/V Cotton rolls	1	244 (2–6) conflicting information 41:69 conflicting information	HVGIC: 341/270 GIC: 387/289	ART criteria	**HVGIC:** 17.8%*/17.8%/17.8% **GIC:** 26.6%*/26.6%/26.6%	NR
Olegario et al., (2019)	GIC (Equia Fil) C (Dyract) GC (CAR-glass)	Class I Class II Cotton rolls	3	568 (4–8) conflicting information 74:76 conflicting information	GIC: 80/78 Class I 93/92 Class II C: 86/84 Class I 93/92 Class II GC: 115/112 Class I 101/100 Class II	Retention, wear, marginal adaptation, secondary caries, pulp inflammation, tooth extraction	**GIC:** Cumulative 43.8%/14.6%/17.5% Class I 17%*/5.7%/6.0%† Class II 44%*/14.7%/17.6%† **C:** Cumulative 43.8%/14.6%/17.5% Class I 22%*/7.3%/8%† Class II 44%*/14.7%/17.6%† **GC:** Cumulative 63.7%/21.2%/28.7% Class I 38%*/12.7%/14.7%† Class II 64%*/21.3%/28.9%†	NR
Olegario et al., (2020)	HVGIC^1^ (Fuji IX Gold Label) HVGIC^2^ (Vitro Molar) HVGIC^3^ (Maxxion R)	Class I Cotton rolls	2	150 (4–8) 74:76	HVGIC^1^: 49/44 HVGIC^2^: 54/50 HVGIC^3^: 47/41	Retention, wear, marginal adaptation, secondary caries, pulp inflammation, tooth extraction	**HVGIC**^**1**^**:** 27.3%*/13.6%/14.7%† **HVGIC**^**2**^**:** 53.5%*/26.8%/31.8%† **HVGIC**^**3**^**:** 60.4%*/30.2%/37.1%†	**HVGIC**^**1**^**:** SC:NR MA: 8% F: NR LR: 16% **HVGIC**^**2**^**:** SC: 4% MA: 11% F: NR LR: 33% **HVGIC**^**3**^**:** SC: NR MA: 8.5% F: NR LR: 44.7%
Oliveira et al., (2008)	CR (TPH Spectrum)	Class I Rubber dam	1.5	32 (4–10) 15:17	Bevel: 47/38 No bevel: 47/38	Modified USPHS	**Bevel:** 2.63%/1.75%/1.8% **No bevel:** 5.26%/3.51%/3.5%	**Bevel:** SC: 2.13% MA: 0% F:NR LR:NR **No Bevel:** SC: 4.26% MA: 0% F:NR LR:NR
Santamaria et al., (2017)	C (Dyract)	Class II NR	2.5	169 (3–8) NR	C: 65/58	Success major failure, minor failure	**C:** 32.8%*/13.12%/14.7%†	**C:** SC:15%* MA:NR F/LR: 9%*
Welbury et al., (1991)	A (Amalcap) GIC (Ketac Fill)	Class I, II Cotton rolls	5	76 (5–11) NR	A: 119/119 GIC: 119/119	Modified USPHS	**A:** 20.2%*/4%/4.4%† **GIC:** 32.8%*/6.6%/7.6%†	**A:** SC: 9.2% MA:NR F: 4.2% LR: 3.4% **GIC:** SC: 5.9% MA:NR F: 5.0% LR:14.3%
Yassen, (2009)	GIC (Ionofil) with conditioner (GC Cavity Conditioner) GIC (Ionofil) without conditioner	Class I Cotton rolls	1	96 (6–7) NR	GIC with conditioner: 48/39 GIC without conditioner: 48/39	ART criteria	**GIC with conditioner:** 26%*/26%/26% **GIC without conditioner:** 33%*/33%/33%	**GIC with conditioner:** SC: NR MA: 5%* F: NR LR: 16%* **GIC without conditioner:** SC: NR MA: 8%* F: NR LR: 20%*

*RCT* randomised controlled trial, *A* amalgam, *ART* Atraumatic restorative treatment, *BFCR* bulk-fill composite resin, *C* compomer, *CR* composite resin, *CSC* composite strip crown, *ER* etch and rinse, *F* fracture, *GC* glass carbomer, *GIC* glass-ionomer cement, *HT* Hall technique, *HVGIC* high-viscosity glass-ionomer cement,, *LR* loss of retention, *MA* marginal adaptation, *MI* marginal integrity, *MRGIC* metal-reinforced glass-ionomer cement, *NR* not reported, *NRCT* non-restorative caries treatment, *RMGIC* resin-modified glass-ionomer cement, *SC* secondary caries, *SE* self-etch, *ZC* zirconia crown, * according to the authors (information written by authors of the trial in the paper), ** based on the sum of “Charly” criteria, † results based on Kaplan–Meier statistics, § evaluation by means of photographs and/or radiographs, ‡ per protocol analysis

## Discussion

This systematic review aimed to investigate the quality of the evidence of published RCTs on the clinical effectiveness of contemporary restorative materials in vital primary teeth.

Restorative materials used covered a wide spectrum, being amalgam, different glass-ionomer cements, glass carbomer, compomer, composite resins and preformed crowns. Failure rates of the different materials varied greatly between the studies, as Chisini et al. 2018 also concluded (Chisini et al. 2018). Glass-ionomer cements demonstrated 7.6–29.9%, among them high-viscosity and the metal-reinforced glass-ionomer cements exhibiting the highest failure rates—the later agreeing with existing literature (Chisini et al. 2018). Amalgam restorations reached up to similar high failure rates (1–28%) with glass-ionomer cements. Glass carbomer restorations showed up to 46.2% failure rate; however, only one study with this material was included in the review. Composite resin restorations showed a failure rate of 0–19.5%, being slightly higher than that reported in a previous review (1.7–12.9%) (Chisini et al. 2018). However, included studies differed from the present review, as studies without control group were included in the first one and a language and year of publication restriction was imposed by those authors. The lowest failure rates in our study was shown by GIC, RMGIC and compomers and not by composite resin restorations as in Chisini et al. 2018 (Chisini et al. 2018). Regarding paediatric crowns, similar low failure rates (3.1%) are reported for PMC in the previous literature (Chisini et al. 2018), which does not include zirconia or composite strip crowns, with failure rates in our study of 3.3% and 15% accordingly. However, due to the fact that composite resin accumulates more plaque than zirconia (Alaki et al. 2020) or metal (Adamczyk and Spiechowicz 1990), the higher failure rate of composite strip crowns are justified. It has to be taken into consideration that composite strip crowns can also be classified as multi-surface composite resin restorations. Strictly speaking, strip crowns support the forming of the crown and the restorative material they are filled with decides about the long-term outcome. A systematic review by Alrashdi et al. (2021) showed that zirconia crowns may be an acceptable alternative to PMC showing similar retention rate, fracture resistance, and gingival health parameters (Alrashdi et al. 2021). The results of this systematic review do not allow for a direct comparison of crown failure rates due to the fact that PMCs were placed on primary molars and zirconia crowns on primary incisors. Especially for aesthetic crowns, the extent of the carious lesion may influence the long-term success, as adhesively bonded restorations require a maximum of bonded surface area. Most of the published studies with crowns report on crown placement after pulp therapy and their success may be influenced more by the management of the pulp treatment, rather than the crown itself (Alrashdi et al. 2021). In our review, 2 included studies examined PMC, zirconia or composite strip crowns without prior pulp therapy (Alaki et al. 2020; Santamaria et al. 2017). Based on the small number of included studies and the high risk of bias no certain conclusions can be drawn.

Some studies investigated the type of the restorative material used in various cavity designs, among them mostly on Class-I or Class-II cavities. However, information on the comparison for the same restorative materials in different cavity classes was limited. In general, literature agrees with the results of the present review that Class-I restorations tend to have lower failure rates than Class-II (Chisini et al. 2018).

The restorative materials used for ART were evaluated separately from their use for the conventional restorative approach. As cavity preparation and caries removal are performed with hand instruments for ART, the cavity design and extent of caries removal differ from the conventional restorative approach, where rotary instruments are used. Both may affect the outcome of the restorative treatment. When ART was examined—with most studies placing glass-ionomer cement restorations after caries excavation—the failure rates climbed up to 37.1%, which complies with the Cochrane Review about ART restorations, suggesting that ART shows higher failure rates than conventional high-viscosity glass-ionomer cement restorations (Dorri et al. 2017). Given the higher probability of failure when glass-ionomer cements are applied by ART, this treatment approach may be limited to situations, where rotary cavity preparation is not available or where Class-I cavities need to be restored with a HVGIC in small children with limited compliance.

Apart from the 10 ART studies (Table 3 and 4), only 10 out of 19 studies gave information on the involved caries excavation technique (complete (Alves dos Santos et al. 2010; Cavalheiro et al. 2020; Ehlers et al. 2019; Hse and Wei 1997; Kavvadia et al. 2004; Kilpatrick et al. 1995; Lenzi et al. 2017; Santamaria et al.^2017^; Welbury et al. 1991) vs. selective (Alves dos Santos et al. 2010; Kavvadia et al. 2004; Kupietzky et al. 2019; Santamaria et al. 2017)). First and foremost, comparison of heterogenic studies would not result to a valid conclusion. Second, since some of the USPHS criteria can be related to residual dentine caries, e. g. hypersensitivity, lack of this information could potentially influence the result of the study and attribute it to the material rather than to the excavation technique. Therefore, it should be suggested that future clinical studies should include more extensive information regarding the implemented procedures.

The level of experience and the number of operators as well as the setting of the clinical study were considerably inconsistent among the 29 included studies. The number of operators was 1–12, usually 1–2 and in some cases, it was not reported at all. Experience levels started from final year undergraduate students and went up to postgraduate students and specialized dentists, although this may have a tremendous influence on the clinical outcome. The exact years of experience of dentists are not mentioned in any study. Study setting for most ART studies was university/dental school type, although this technique is not originally designed for this. Most of the included studies for all other materials were performed in dental colleges and only a few in private practices. All those factors cause diversities within the included studies which could potentially influence their results and thus their comparison.

There was no uniform adhesion technique chosen for composite resin placement. Most of the studies with composite resin restorations (8/12) chose the etch-and-rinse procedure (Akman and Tosun 2020; Alves dos Santos et al. 2010; Barr-Agholme et al. 1991; Cavalheiro et al. 2020; Dermata et al. 2018; El-Housseiny et al. 2019; Hse and Wei 1997; Kupietzky et al. 2019; Oliveira et al. 2008). This makes comparisons between the studies challenging, even if the same restorative material is used. Only one study used the same adhesive in both etch-and-rinse and self-etching mode and was able to directly compare their effect (Lenzi et al. 2017).

With respect to the heterogeneity and the risk of bias of the included studies, only 4 studies out of 29 were rated as having “some concerns” in the quality assessment for the potential risk of bias (Table 2). A quantitative synthesis could not be performed because of the heterogeneity in study designs, comparisons chosen, outcomes described, and the overall high risk of bias of included RCTs. Accordingly, and despite the number of 29 included studies, clear recommendations for clinical practice based on pooling effects from the available evidence were impossible to be drawn.

Compared to Schwendicke et al. 2016, where a review and network meta-analysis was performed, the rationale of this systematic review was different (Schwendicke et al. 2016). The authors of the aforementioned study limited the publication years (2005–2015) and performed a meta-analysis by including “high risk of bias” papers. As a matter of fact, it is questionable if a meta-analysis is meaningful, when high risk of bias is identified and unclear conclusions are drawn.

The quality of evidence for the restorative materials evaluated at a minimum of 12 months varied and depended on factors, such as sample size calculation, randomisation, allocation concealment, blinding technique and dropout rate (Table 2 and in Fig. 2). Randomisation was a conflicting issue, since few authors presented detailed information and allocation concealment was not described thoroughly, if not at all. Based on the nature of the dental procedures and the different handling of different restorative materials, it is to be expected that double-blinding is difficult—if not impossible—to be achieved. Nevertheless, some studies attempted to minimise performance bias by excluding the operator(s) from assessing the effect of interventions. Moreover, time of physiological tooth exfoliation is not known, and is especially important for older children or studies with a wide age range. Therefore, despite the fact that factor age of the child may be involved in the success rates and longevity of the restorations placed, its effect remains uncertain.

## Strengths

One of the major strengths of the current review is its stringent inclusion criteria—RCTs with more than 40 restorations per group, with a minimum of 12-month follow-up duration and less than 30% drop-out rate. In contrast to the latest published study on the same subject (Chisini et al. 2018), studies with no control group were excluded as well as when the control group was not standardised (Innes et al. 2011). Only studies with a minimum of 40 restorations per group were included, to comply with recent literature (Chisini et al. 2018) and in cases where at least one group had less than 40 restorations, the study was excluded (Andersson-Wenckert and Sunnegardh-Gronberg 2006). Drop-out ratio was set to a maximum of 30% either at patient level or at tooth level, to make included studies comparable (Tedesco et al. 2017). Drop-out ratios were either provided by the authors of each paper, or calculated at patient level and at tooth level according to the information given. Exfoliated teeth—either mentioned in the text or calculated by the authors—were not included in the drop-out. However, if another publication of the same cohort with a shorter follow-up met the aforementioned criteria, it was considered eligible for inclusion. The exclusive inclusion of randomised controlled clinical trials (RCTs) aimed to diminish the risk of selection bias, as restorative materials were randomly allocated among treatment groups (Schwendicke et al. 2016). Standardising the calculation of the failure rates reported in each trial and calculating the annual failure rate for all restorative procedures of the included papers (Opdam et al. 2014) allowed for direct comparisons between the included studies, offering another great strength. As opposed to other reviews, there was no restriction in the year of publication of included literature, as well as no language restriction.

## Limitations

The large number of restorative materials evaluated across the studies, the difference in the study design (split-mouth vs. parallel), as well as their different levels of risk of bias, did not allow for studies to be included in the meta-analysis and that could be regarded as the major limitation of the systematics review. Regarding glass carbomer restorations, only one study was included in the review. Studies with longer observation periods than 12-month set were scarce and high drop-out rates along with a high number of exfoliations occurring in primary dentition did not allow reporting for longer follow-up periods. Despite the fact that the 12-month follow-up was chosen to be in accordance with previous reviews, the number of studies was small and thus the review does not report on failure rates of restorative material in vital primary teeth for longer time periods.

Another limitation is that 70% of the included ART studies were performed in a school setting and 30% at university, although this technique is originally designed to be conducted under field conditions without access to electricity (Frencken 2009). Apart from material characteristics, the choice of the restorative technique and the setting may influence the outcome, which was confirmed by the variation of AFRs reported in the included ART studies. For ART, the caries removal with hand instruments along with the restoration under field conditions (Frencken and Holmgren 1999) may be influential factors for the longevity of the restoration.

The procedure of caries excavation was not evaluated as a parameter, since information in the included papers was limited. Nevertheless, the extent of caries removal is of relevance for the success of the restorative treatment, as it was shown that composite resin restorations in primary molars presented a higher failure rate when selective caries removal was performed compared to complete caries removal, although the former technique was associated with a better outcome for the pulp (Liberman et al. 2020).

No universal study design (split mouth vs. parallel arm) was met, having an effect on quality of evidence. In split-mouth designs each patient acts as his own control which reduces inter-subject variability and/or random error, thus increasing study accuracy and power. Moreover, parameters which can cause confusion in the level of bias—such as age, oral hygiene level, and general health—can be eliminated in split-mouth design. The so-called “carry-across effect” is a major limitation of this study design describing distortion of the treatment effect due to mutual influence of both treatment sides (Pozos-Guillen et al. 2017). In cases of restorative treatment, however, this is unlikely to happen, since each material is placed on a certain tooth. However, a split-mouth design is contraindicated in case of asymmetrical distribution of carious lesions (e. g. varying severity) within the oral cavity (Pozos-Guillen et al. 2017).

Since caries risk was reported in very few studies, and there was no distinction between the sides or quadrants of the mouth, selection of the specific study design should be reconsidered. In addition, eligible patients for split-mouth set ups are more difficult to recruit, since symmetry in appearance of the disease appears more rarely in patients’ mouths. However, non-eligibility of a large number of patients imposes restrictions in the ability to generalize the conclusions of the studies, since they may not apply to the public. For example, choosing patients with cavities in all quadrants might create bias towards patients with a higher total number of cavities in their oral cavity (Chaffee et al. 2017), which is associated with the socioeconomic status (Schwendicke et al. 2015). On the other hand, one major aspect of parallel group studies is randomization and allocation concealment, which in the present review was the most common factor for leveling risk of bias to high risk. This study design also increases the number of subjects to be recruited. However, their statistical analysis is simpler than the one of the split-mouth studies. Other limitations could be factors that may cause diversities, such as the number of operators (1–12), experience level (final year undergraduate students up to specialized dentists) and study setting (university vs. private practice). It is often discussed that response for authors is very limited in systematic reviews, as reported by EAPD review group 1. Due to this experience, we decided not to contact the authors for further information. This also has to be accounted as a limitation of the current review.

## Conclusions

Considering any limitations of the present review, the following conclusions can be made:

- The included RCTs were mainly evaluated as at high-risk of bias, with the domains of randomisation and allocation concealment being the most problematic.
- The results ranged within the same material group, as different evaluation criteria were used among the studies.
- In view of phasing down the use of dental amalgam, this restorative material may no longer be recommended in primary dentition given the toxicological concerns and available tooth-coloured restorative materials with lower AFRs.
- The type of glass-ionomer cement plays an important role on clinical outcome: Due to the inferior performance, MRGIC and HVGIC cannot be recommended. RMGIC may be a treatment option for Class-I and Class-II restorations in primary molars.
- Compomers, hybrid composite resins and bulk-fill composite resins demonstrated similar failure rates, with compomers exhibiting the lowest AFR among them.
- Crown material (zirconia or preformed metal) may have an impact on failure rates, although the existing data are extremely limited.
- Restorative materials used for ART reported comparable AFRs to conventional amalgam restorations.
- All in all, future thoroughly implemented RCTs evaluating restorations in primary teeth are needed to draft clear recommendations for the best restorative treatment approach.

## Supplementary Information

Below is the link to the electronic supplementary material.

Supplementary file1 (DOCX 16 KB)

Supplementary file2 (DOCX 16 KB)

Supplementary file3 (DOCX 8 KB)

Supplementary file4 (DOCX 8 KB)

Supplementary file5 (DOCX 8 KB)
