# Supplementary material for: Clinical effectiveness of restorative materials for the restoration of carious primary teeth without pulp therapy: a systematic review
Source: Eur Arch Paediatr Dent. 2022 Jul 12;23(5):727–59. doi: 10.1007/s40368-022-00725-7 (PMC9637592; doi:10.1007/s40368-022-00725-7)
Supplement: Supplementary file 1 — Supplementary file1 (DOCX 16 KB) [file 40368_2022_725_MOESM1_ESM.docx]

| Clinical effectiveness of restorative materials including new biomaterials for the restoration of carious primary teeth. | | | |
| --- | --- | --- | --- |
| **Search number** | **Query** | **Filters** | **Results** |
| **1** | (primary[Title/Abstract] OR baby[Title/Abstract] OR deciduous[Title/Abstract] OR milk[Title/Abstract]) AND (tooth[Title/Abstract] OR teeth[Title/Abstract] OR dental[Title/Abstract] OR dentition[Title/Abstract]) |  | 29.204 |
| **2** | (restorati*[Title/Abstract] OR crown[Title/Abstract] OR filling*[Title/Abstract]) AND (cari*[Title/Abstract] OR decay[Title/Abstract] OR cavities[Title/Abstract] OR dentine[Title/Abstract] OR lesion[Title/Abstract]) |  | 17.1 |
| **3** | ((restorati*[Title/Abstract] OR crown[Title/Abstract] OR filling*[Title/Abstract]) AND (cari*[Title/Abstract] OR decay[Title/Abstract] OR cavities[Title/Abstract] OR dentine[Title/Abstract] OR lesion[Title/Abstract])) AND ((primary[Title/Abstract] OR baby[Title/Abstract] OR deciduous[Title/Abstract] OR milk[Title/Abstract]) AND (tooth[Title/Abstract] OR teeth[Title/Abstract] OR dental[Title/Abstract] OR dentition[Title/Abstract])) |  | 1.778 |
| **4** | glass[Title/Abstract] OR polyalkenoate[Title/Abstract] OR ionomer[Title/Abstract] OR cement*[Title/Abstract] OR resin*[Title/Abstract] OR metal[Title/Abstract] OR composite*[Title/Abstract] OR amalgam[Title/Abstract] OR compomer*[Title/Abstract] OR Polyacid[Title/Abstract] OR biomaterial[Title/Abstract] OR bio-active[Title/Abstract] |  | 673.87 |
| **5** | (glass[Title/Abstract] OR polyalkenoate[Title/Abstract] OR ionomer[Title/Abstract] OR cement*[Title/Abstract] OR resin*[Title/Abstract] OR metal[Title/Abstract] OR composite*[Title/Abstract] OR amalgam[Title/Abstract] OR compomer*[Title/Abstract] OR Polyacid[Title/Abstract] OR biomaterial[Title/Abstract] OR bio-active[Title/Abstract]) AND (((restorati*[Title/Abstract] OR crown[Title/Abstract] OR filling*[Title/Abstract]) AND (cari*[Title/Abstract] OR decay[Title/Abstract] OR cavities[Title/Abstract] OR dentine[Title/Abstract] OR lesion[Title/Abstract])) AND ((primary[Title/Abstract] OR baby[Title/Abstract] OR deciduous[Title/Abstract] OR milk[Title/Abstract]) AND (tooth[Title/Abstract] OR teeth[Title/Abstract] OR dental[Title/Abstract] OR dentition[Title/Abstract]))) |  | 856 |
| **6** | (glass[Title/Abstract] OR polyalkenoate[Title/Abstract] OR ionomer[Title/Abstract] OR cement*[Title/Abstract] OR resin*[Title/Abstract] OR metal[Title/Abstract] OR composite*[Title/Abstract] OR amalgam[Title/Abstract] OR compomer*[Title/Abstract] OR Polyacid[Title/Abstract] OR biomaterial[Title/Abstract] OR bio-active[Title/Abstract]) AND (((restorati*[Title/Abstract] OR crown[Title/Abstract] OR filling*[Title/Abstract]) AND (cari*[Title/Abstract] OR decay[Title/Abstract] OR cavities[Title/Abstract] OR dentine[Title/Abstract] OR lesion[Title/Abstract])) AND ((primary[Title/Abstract] OR baby[Title/Abstract] OR deciduous[Title/Abstract] OR milk[Title/Abstract]) AND (tooth[Title/Abstract] OR teeth[Title/Abstract] OR dental[Title/Abstract] OR dentition[Title/Abstract]))) | Humans | 689 |
| **7** | (primary teeth[MeSH Terms]) AND (glass[Title/Abstract] OR polyalkenoate[Title/Abstract] OR ionomer[Title/Abstract] OR cement*[Title/Abstract] OR resin*[Title/Abstract] OR metal[Title/Abstract] OR composite*[Title/Abstract] OR amalgam[Title/Abstract] OR compomer*[Title/Abstract] OR Polyacid[Title/Abstract] OR biomaterial[Title/Abstract] OR bio-active[Title/Abstract]) |  | 1.49 |
| **8** | (primary teeth[MeSH Terms]) AND (glass[Title/Abstract] OR polyalkenoate[Title/Abstract] OR ionomer[Title/Abstract] OR cement*[Title/Abstract] OR resin*[Title/Abstract] OR metal[Title/Abstract] OR composite*[Title/Abstract] OR amalgam[Title/Abstract] OR compomer*[Title/Abstract] OR Polyacid[Title/Abstract] OR biomaterial[Title/Abstract] OR bio-active[Title/Abstract]) | Humans | 1.417 |
| **9** | ((primary teeth[MeSH Terms]) AND (glass[Title/Abstract] OR polyalkenoate[Title/Abstract] OR ionomer[Title/Abstract] OR cement*[Title/Abstract] OR resin*[Title/Abstract] OR metal[Title/Abstract] OR composite*[Title/Abstract] OR amalgam[Title/Abstract] OR compomer*[Title/Abstract] OR Polyacid[Title/Abstract] OR biomaterial[Title/Abstract] OR bio-active[Title/Abstract]) AND (humans[Filter])) AND (((restorati*[Title/Abstract] OR crown[Title/Abstract] OR filling*[Title/Abstract]) AND (cari*[Title/Abstract] OR decay[Title/Abstract] OR cavities[Title/Abstract] OR dentine[Title/Abstract] OR lesion[Title/Abstract])) AND ((primary[Title/Abstract] OR baby[Title/Abstract] OR deciduous[Title/Abstract] OR milk[Title/Abstract]) AND (tooth[Title/Abstract] OR teeth[Title/Abstract] OR dental[Title/Abstract] OR dentition[Title/Abstract]))) |  | 414 |
| **10** | ((dental materials[MeSH Terms]) AND (primary teeth[MeSH Terms])) OR (deciduous dentition[MeSH Terms]) |  | 12.496 |
| **11** | (((dental materials[MeSH Terms]) AND (primary teeth[MeSH Terms])) OR (deciduous dentition[MeSH Terms])) AND (((restorati*[Title/Abstract] OR crown[Title/Abstract] OR filling*[Title/Abstract]) AND (cari*[Title/Abstract] OR decay[Title/Abstract] OR cavities[Title/Abstract] OR dentine[Title/Abstract] OR lesion[Title/Abstract])) |  | 881 |
| **12** | (((dental materials[MeSH Terms]) AND (primary teeth[MeSH Terms])) OR (deciduous dentition[MeSH Terms])) AND (((restorati*[Title/Abstract] OR crown[Title/Abstract] OR filling*[Title/Abstract]) AND (cari*[Title/Abstract] OR decay[Title/Abstract] OR cavities[Title/Abstract] OR dentine[Title/Abstract] OR lesion[Title/Abstract])) | Humans | 867 |
| **13** | (((dental materials[MeSH Terms]) AND (primary teeth[MeSH Terms])) OR (deciduous dentition[MeSH Terms])) AND (((restorati*[Title/Abstract] OR crown[Title/Abstract] OR filling*[Title/Abstract]) AND (cari*[Title/Abstract] OR decay[Title/Abstract] OR cavities[Title/Abstract] OR dentine[Title/Abstract] OR lesion[Title/Abstract])) | Randomized Controlled Trial, Humans | 154 |
| **14** | (((dental materials[MeSH Terms]) AND (primary teeth[MeSH Terms])) OR (deciduous dentition[MeSH Terms])) AND (((restorati*[Title/Abstract] OR crown[Title/Abstract] OR filling*[Title/Abstract]) AND (cari*[Title/Abstract] OR decay[Title/Abstract] OR cavities[Title/Abstract] OR dentine[Title/Abstract] OR lesion[Title/Abstract])) | Randomized Controlled Trial | 155 |
| **15** | ("glass"[Title/Abstract] OR "polyalkenoate"[Title/Abstract] OR "ionomer"[Title/Abstract] OR "cement*"[Title/Abstract] OR "resin*"[Title/Abstract] OR "metal"[Title/Abstract] OR "composite*"[Title/Abstract] OR "amalgam"[Title/Abstract] OR "compomer*"[Title/Abstract] OR "Polyacid"[Title/Abstract] OR "biomaterial"[Title/Abstract] OR "bio-active"[Title/Abstract]) AND (("restorati*"[Title/Abstract] OR "crown"[Title/Abstract] OR "filling*"[Title/Abstract]) AND ("cari*"[Title/Abstract] OR "decay"[Title/Abstract] OR "cavities"[Title/Abstract] OR "dentine"[Title/Abstract] OR "lesion"[Title/Abstract]) AND (("primary"[Title/Abstract] OR "baby"[Title/Abstract] OR "deciduous"[Title/Abstract] OR "milk"[Title/Abstract]) AND ("tooth"[Title/Abstract] OR "teeth"[Title/Abstract] OR "dental"[Title/Abstract] OR "dentition"[Title/Abstract]))) AND ("control*"[All Fields] OR "random*"[All Fields]) |  | 410 |
| **16** | (("dental materials"[MeSH Terms] AND "tooth, deciduous"[MeSH Terms]) OR "tooth, deciduous"[MeSH Terms]) AND (("restorati*"[Title/Abstract] OR "crown"[Title/Abstract] OR "filling*"[Title/Abstract]) AND ("cari*"[Title/Abstract] OR "decay"[Title/Abstract] OR "cavities"[Title/Abstract] OR "dentine"[Title/Abstract] OR "lesion"[Title/Abstract])) AND ("control*"[All Fields] OR "random*"[All Fields]) |  | 410 |
