# Supplementary material for: Clinical effectiveness of restorative materials for the restoration of carious primary teeth without pulp therapy: a systematic review
Source: Eur Arch Paediatr Dent. 2022 Jul 12;23(5):727–59. doi: 10.1007/s40368-022-00725-7 (PMC9637592; doi:10.1007/s40368-022-00725-7)
Supplement: Supplementary file 3 — Supplementary file3 (DOCX 8 KB) [file 40368_2022_725_MOESM3_ESM.docx]

Embase via Ovid search, 28.12.2020

|  | | |
| --- | --- | --- |
| 1 | ((glass or polyalkenoate or ionomer or cement* or resin* or metal or composite* or amalgam or compomer* or Polyacid or biomaterial or bio-active) and (primary or milk or baby or deciduous)).ab. | 71166 |
| 2 | (caries or decay or cavities or lesion).ab. | 688715 |
| 3 | 1 and 2 | 5570 |
| 4 | (restorative or restoration or crown or filling).ab. | 269167 |
| 5 | 3 and 4 | 720 |
