# Supplementary material for: Clinical effectiveness of restorative materials for the restoration of carious primary teeth without pulp therapy: a systematic review
Source: Eur Arch Paediatr Dent. 2022 Jul 12;23(5):727–59. doi: 10.1007/s40368-022-00725-7 (PMC9637592; doi:10.1007/s40368-022-00725-7)
Supplement: Supplementary file 4 — Supplementary file4 (DOCX 8 KB) [file 40368_2022_725_MOESM4_ESM.docx]

Cochrane Library search, 28.12.2020

#1 (glass or polyalkenoate or ionomer or cement* or resin* or metal or composite* or amalgam or compomer* or Polyacid or biomaterial or bio-active):ti,ab,kw (Word variations have been searched) 38813

#2 (glass or polyalkenoate or ionomer or cement* or resin* or metal or composite* or amalgam or compomer* or Polyacid or biomaterial or bio-active):ti,ab,kw AND (primary OR milk OR baby OR deciduous):ti,ab,kw (Word variations have been searched) 18645

#3 (glass or polyalkenoate or ionomer or cement* or resin* or metal or composite* or amalgam or compomer* or Polyacid or biomaterial or bio-active):ti,ab,kw AND (primary OR milk OR baby OR deciduous):ti,ab,kw AND (tooth OR teeth OR molar OR dental):ti,ab,kw (Word variations have been searched) 1677

#4 (glass or polyalkenoate or ionomer or cement* or resin* or metal or composite* or amalgam or compomer* or Polyacid or biomaterial or bio-active):ti,ab,kw AND (primary OR milk OR baby OR deciduous):ti,ab,kw AND (tooth OR teeth OR molar OR dental):ti,ab,kw AND (caries OR decay OR cavities):ti,ab,kw (Word variations have been searched) 944
