# Supplementary material for: Clinical effectiveness of restorative materials for the restoration of carious primary teeth without pulp therapy: a systematic review
Source: Eur Arch Paediatr Dent. 2022 Jul 12;23(5):727–59. doi: 10.1007/s40368-022-00725-7 (PMC9637592; doi:10.1007/s40368-022-00725-7)
Supplement: Supplementary file 5 — Supplementary file5 (DOCX 8 KB) [file 40368_2022_725_MOESM5_ESM.docx]

LILACS search, 28.12.2020

primary OR milk OR baby OR deciduous [Abstract words] and restoration OR restorative OR crown OR filling [Words] 484

primary OR milk OR baby OR deciduous [Abstract words] and restoration OR restorative OR crown OR filling [Abstract words] 395

primary OR milk OR baby OR deciduous [Abstract words] and glass OR polyalkenoate OR ionomer OR cement* OR resin* OR metal OR composite* OR amalgam OR compomer* OR Polyacid OR biomaterial OR bio-active [Words] 0

primary OR milk OR baby OR deciduous [Abstract words] and restoration OR restorative OR crown OR filling [Abstract words] and caries OR cavities OR decay [Abstract words] 107

primary OR milk OR baby OR deciduous [Abstract words] and restoration OR restorative OR crown OR filling [Abstract words] and dental materials [Words] 8
